# Biological toxicities as surrogate markers of efficacy in patients treated with mTOR inhibitors for metastatic renal cell carcinoma

**DOI:** 10.1186/s12885-016-2993-7

**Published:** 2017-01-06

**Authors:** M. Jebali, R. Elaidi, M. Brizard, J. Fouque, C. Takouchop, B. Sabatier, S. Oudard, J. Medioni

**Affiliations:** 1 Department of Medical Oncology (Centre d’Essais Clinique Précoces en Cancérologie [CEPEC]), Hôpital Européen Georges Pompidou, 20 rue Leblanc, 75015 Paris, France; 2 Association pour la Recherche sur les Thérapeutiques Innovantes en Cancérologie (ARTIC), Hôpital Européen Georges Pompidou, Paris, France; 3 Department of Pharmacy, Hôpital Européen Georges Pompidou, Paris, France; 4 Paris Descartes University, Paris, France

**Keywords:** Drug-related side effects, Adverse reactions, mTOR inhibitors, Metastatic renal cell carcinoma, Biomarkers

## Abstract

**Background:**

Metabolic toxicities of mTOR inhibitors (mTORi) are well characterized. The purpose of the study was to investigate the relationship between these metabolic toxicities and mTORi efficacy.

**Methods:**

From 2007 to 2011, metabolic toxicities were retrospectively collected in patients treated with an mTORi (everolimus, temsirolimus) for a metastatic renal cell carcinoma (mRCC) in a single institution. Patients were eligible if they have received an mTORi for at least 28 days. Changes in the following parameters were analyzed: lymphocytes, serum creatinine, glycemia, serum phosphate, liver transaminases, cholesterol, and triglycerides. The efficacy was assessed by progression-free survival (PFS) and tumor response.

**Results:**

Data were collected from seventy-five patients (everolimus = 44 patients; temsirolimus = 31 patients). Six patients exhibited a partial response, 42 a stable disease and 15 had a progressive disease (12 missing). After a median follow-up of 12.8 months, the median PFS was 6.7 months (95% confidence interval: 4.0-9.1 months). Patients with CB had a statistically more severe absolute increase of glycemia and absolute decrease in phosphatemia (*p* = 0.002 and *p* = 0.02 respectively). The Progression Free Survival was significantly higher with the onset rate of hypophosphatemia (*p* = 0.03) and hyperglycemia (*p* = 0.001) and lower with the onset rate of lymphopenia (*p* = 0.004).

**Conclusions:**

Hyperglycemia, hypophosphatemia and lymphopenia, were significantly associated with tumor response and/or PFS. Those events, as well as their onset rate, should be prospectively monitored as predictors of response to mTORi.

## Background

Angiogenesis plays an important role in the invasion and dissemination of renal cell carcinoma (RCC), and dysregulation of genes encoding for the vascular endothelial growth factor (VEGF) and its receptors (VEGF-R) is the mainstay of this process [1]. Molecules targeting VEGF and VEGF-R have been developed. Tyrosine kinase inhibitors (TKI) (sunitinib, sorafenib, axitinib, cabozantinib) and monoclonal antibodies (bevacizumab) are routinely used as first- or second-line therapy [2–6].

The mammalian target of rapamycin (mTOR) pathway is downstream of the phosphoinositide 3-kinase and AKT pathway. Increased levels of phospho-AKT have been observed in RCC [7]. Therefore, the inhibition of the mTOR pathway can potentially inhibit the tumor progression. In phase III controlled trials, temsirolimus and everolimus have shown antitumoral efficacy in patients with mRCC. Temsirolimus was compared in first-line treatment with interferon-α, showing a statistically significant benefit in favor of temsirolimus for progression-free survival (PFS) and overall survival (OS) [8]. In a phase III trial against placebo (RECORD-1), everolimus was evaluated in patients with progressive disease after TKI failure resulting in a clear benefit in PFS, but not in OS [9]. Those results led to an approved use of temsirolimus and everolimus in mRCC. All of these targeted therapies are responsible for toxicities. Some of them are common for all of antiangiogenic therapy such as fatigue, anorexia, diarrhea and anemia whereas other adverse events are class or agent specific [3–9]. With mTORi, the most frequent clinical adverse events reported in phase III trials were skin rash, nausea, mucositis, and diarrhea [8, 9]. A class-specific pulmonary toxicity is observed in 35% of patients receiving an mTORi, going from cough to irreversible interstitial pneumonitis [10]. The most common metabolic toxicities were anemia, hypercholesterolemia, hypertriglyceridemia, increase in serum creatinine, and lymphopenia [8, 9].

A relationship between toxicities of antiangiogenics and their efficacy has been extensively studied. For instance, hypertension and hypothyroidism have been shown to be associated with a better disease outcome in patients receiving sunitinib or bevacizumab [11–17]. As regards mTORi, a relationship has been found between the occurrence of pneumonitis and a better clinical outcome for both everolimus and temsirolimus [18].

Some clinical toxicities have been shown to be correlated with efficacy of antiangiogenics and mTORi. For mTORi, some of these toxicities were directly related to the inhibition of the mTOR pathways, both in normal and tumoral tissue. Therefore, it has been proposed to investigate toxicity as a surrogate marker for mTORi efficacy [19]. The purpose of the present study was to evaluate retrospectively the relationship between metabolic toxicities and mTORi efficacy in mRCC patients.

## Methods

### Patient population

A retrospective study was conducted in single French institution (Hôpital européen Georges Pompidou, Paris, France) involving mRCC patients, treated with everolimus or temsirolimus from March 2007 to December 2011. All the treated patients, who met the following inclusion criteria, were included in the study: mRCC of any histological subtype; treatment with an mTORi for at least 28 days; ECOG performance status (PS) 0–2; grade 0–2 for neutrophils, hemoglobin, platelets, transaminases, and bilirubin; and creatinine clearance > 50 ml/mn. Patients receiving a concurrent antitumoral agent were not eligible.

### Treatment

Temsirolimus and everolimus were administered on a routine practice according to registered regimens. Intravenous temsirolimus was administered at a dose of 25 mg/m^2^ once a week. Everolimus was administered orally at a starting dose of 10 mg per day. The duration of a cycle was 28 days.

According to French guidelines, the occurrence of a grade 4 toxicity led to a definitive interruption of mTORi. In case of grade-3 toxicity, the treatment was discontinued until recovery to grade 1, and then a reduced dose administration was permitted. In case of grade-2 toxicity, the treatment was discontinued until recovery to grade 1, and then resumed at a similar dose administration. In case of grade-1 toxicity, the dose was unchanged.

### Safety assessment

Selected biological parameters (white blood cells, serum creatinine, glycemia, phosphatemia, liver transaminases, cholesterol, and triglycerides) were assessed every 4 weeks. Their baseline value was determined within 7 days before starting the mTOR inhibitor. The most severe toxicity for each selected biological parameter was reported according to the Common Toxicity Criteria for Adverse Events (CTC-AE, version 3.0). The highest absolute change from baseline (*i.e.* difference between baseline value and the worst reported value) was calculated.

### Efficacy assessment

The efficacy assessment was performed according to routine practice every twelve weeks upon Computed Tomography scan or any appropriated imaging method (Magnetic Resonance Imaging, bone scan). The tumoral response was measured using radiological response based on the RECIST 1.1 criteria. The clinical benefit (CB) was defined as the addition of radiological partial response and/or stable disease. The PFS was defined as the time from mTOR initiation to disease progression or last assessment, and OS was defined as the time from treatment start to death or last follow-up.

### Statistical analysis

The primary endpoint was the relation between highest CTC-grade toxicity and tumor response upon RECIST 1.1 criteria. The secondary endpoints were the relation between: time to highest CTC-grade toxicity and tumor response upon RECIST 1.1 criteria; highest absolute change of biological parameters from baseline and tumor response upon RECIST 1.1 criteria; highest absolute change of biological parameters from baseline and PFS; and time to highest CTC-grade toxicity and PFS.

The relation between qualitative variables was evaluated using the chi-square test. The relation between qualitative and quantitative variables was evaluated using the Student’s t-test. Survival data were computed according to the Kaplan-Meier method, categorical data were compared using the log-rank test, and continuous data comparisons were performed using the Cox model. The hazard ratios (HR) were calculated with 95% confidence interval (95% CI). The relations were considered as statistically significant for a *p*-value < 0.05.

## Results

### Patient and treatment characteristics

Data were collected from seventy-five patients. The main characteristics of patients at baseline are summarized in Table 1. The metastatic disease was synchronous of RCC diagnosis in 18 patients, whereas metachronous metastases were observed in 41 patients with a median time to metastases of 23 months. The median number of tumor sites was 1 (range: 1–4).

**Table 1:** Characteristics of the 75 mRCC patients at baseline

Characteristics	
Sex, n (%)	
Male	60 (80)
Female	15 (20)
Age, years	
Median (range)	55 (36–85)
Smokers (current or past), n (%)	36 (48)
Histology, n (%)	
Clear cell carcinoma	56 (74.7)
Papillary cell carcinoma	8 (10.7)
Mixt cell carcinoma	6 (8)
Sarcomatoid cell carcinoma	3 (4)
Bellini carcinoma	1 (1.3)
Chromophobic cell carcinoma	1 (1.3)
Nephrectomy, n (%)	70 (93.3)
Fuhrman grade, n (%)	
1	1 (2)
2	11 (22)
3	25 (50)
4	13 (26)
Missing	25
Primary metastases, n (%)	18 (31)
Missing	17
Bone metastases, n (%)	20 (55.6)
Missing	39
Time to metastases, months	
Median (range)	23 (0.2-118.4)
Missing	35
ECOG-PS, n (%)	
0	24 (32)
≥ 1	51 (68)
Post mTORi anti-cancer treatment	30 (46)
Missing	33

*ECOG-PS* Eastern Cooperative Oncology Group performance status

Forty-four patients (59%) received everolimus for a median duration of 221 days (range: 40 to 838 days), and 31 patients (41%) received temsirolimus for a median duration of 104 days (range: 32 to 504 days). Everolimus was administered as first-line treatment (RECORD-3 study) in 2 patients (4%), as second-line (RECORD-1) in 9 patients (20%), and as third-line or more in 33 patients (66%). Temsirolimus was administered as first-line treatment in 6 patients (19%), as second-line in 11 patients (35%), and as third-line or more in 14 patients (46%).

### Metabolic toxicities

The most frequent all-grade toxicities were lymphopenia, increase in serum creatinine, hypertriglyceridemia, hypercholesterolemia, and hyperglycemia (Table 2). The most frequent grade 3–4 toxicities were lymphopenia and hyperglycemia. Overall, everolimus exhibited a higher rate of toxicity than temsirolimus.

**Table 2:** Metabolic toxicities

Toxicity	Temsirolimus (*n* = 31)		Everolimus (*n* = 44)		Time to highest toxicity grade, days	Highest absolute change from baseline (%)
	All grade n (%)	Grade 3/4 n (%)	All grade n (%)	Grade 3/4 n (%)	Median (range)	Median (range)
Lymphopenia	13 (42)	4 (13)	25 (57)	9 (20)	56 (28; 644)	−31 (−79; 168)
Increase in serum creatinine	14 (45)	-	27 (61)	-	90 (28; 420)	9.5 (−32; 78)
Hyperglycemia	9 (29)	2 (6)	24 (54)	6 (14)	56 (28; 756)	18 (−44; 346)
Hypophosphatemia	2 (6)	1 (3)	11 (25)	3 (7)	56 (28; 168)	−22 (−55; 16)
Increase in ASAT	4 (13)	-	14 (32)	1 (2)	28 (14-; 280)	47 (−75; 552)
Increase in ALAT	2 (6)	-	14 (32)	2 (4)	28 (28; 308)	75 (−68; 1883)
Hypercholesterolemia	13 (42)	-	31 (70)	3(7)	28 (28; 392)	32 (−51; 125)
Hypertriglyceridemia	14 (45)	-	31 (70)	-	56 (14; 224)	85 (−58; 375)

*ASAT* aspartate aminotransferase, *ALAT* alanine aminotransferase

The median time to the highest grade metabolic toxicities ranged between 28 days and 90 days, *i.e.* between the first and the third cycle (Table 2). The earlier toxicities were an increase in liver transaminases and hypercholesterolemia, and the later toxicity was the increase in serum creatinine.

### Efficacy

Twelve patients were not assessable for response because of an early discontinuation related to toxicity. Sixty-three patients were assessable with partial response in 6 patients (9.5%), stable disease in 42 patients (66.7%), and progressive disease in 15 patients (23.8%) (missing data: 12 patients).

After a median follow-up of 12.! months, the median PFS was 6.7 months (median PFS for Clear Cell Carcinoma was 4.8 months, and median PFS for non Clear Cell Carcinoma was 10.2 months (NS)). Fifty-five of 75 patients (73.3%) died of disease, and the median OS was 14 months (median OS for Clear Cell Carcinoma was 14.6 months, and median OS for non Clear Cell Carcinoma was 18 months (NS)).

### Relation between metabolic toxicities and clinical efficacy

#### Tumor response and grade of toxicity

A significant relation was found between CB, and all-grade increase in serum creatinine and liver transaminases. An increase in serum creatinine was found in 92% of patients with CB *vs.* 46% of those with Progressive Disease (PD) (*p* = 0.01); an increase in ASAT was found in 94% of patients with CB *vs.* 66% of those with PD (*p* = 0.04); and an increase in ALAT was found in 100% of patients with CB *vs.* 66% of those with PD (*p* = 0.01). For all other biological parameters, the relation was not significant.

#### Tumor response and highest change in biological parameters as continuous variables

Glycemia increased in CB patients and decreased in PD patients (+61% *vs.* - 5%; *p* = 0.002). Hypophosphatemia was most severe in CB patients compared to PD patients (− 26% *vs.* - 9%; *p* = 0.02). Increase in liver transaminases was larger in CB patients than in PD patients (ASAT: +126% *vs.* +8%, *p* = 0.004; ALAT: +229% *vs.* +4%, *p* = 0.003). For all other biological parameters, the relation was not significant.

#### Tumor response and time to highest grade of toxicity

Median time to grade ≥ 2 lymphopenia was shorter in PD patients than in CB patients (84 days *vs.* median not reached; *p* = 0.01). Median time to grade ≥ 2 hyperglycemia was longer in PD patients than in CB patients (112 *vs.* 56 days; *p* = 0.03). No significant differences were observed in grade-1 toxicities and for the other parameters.

Table 3 summarized relationship between tumor response and toxicity.

**Table 3:** Relation between tumor response and metabolic toxicities

	CTC Grade of Toxicities	Highest change in biological parameters	Median time to CTC Grade ≥ 2 toxicities
	CB (%) / PD (%) (p)	CB (%) / PD (%) (p)	CB (days) / PD (days)
Lymphopenia			NA / 84 (0.01)
Increase in serum creatinine	92 / 46 (0.01)	+61 / -5 (0.002)	
Hyperglycemia			56/112 (0.03)
Hypophosphatemia		−26 / -9 (0.02)	
Increase in ASAT	94 / 66 (0.04)	+126 / +8 (0.004)	
Increase in ALAT	100 / 66 (0.01)	+229 / +4 (0.003)	
Hypercholesterolemia			
Hypertriglyceridemia			

*CB* Clinical Benefit, *PD* Progressive Disease, *NA* not attained

#### Tumor response and time to toxicity

In PD patients compared with CB patients, lymphopenia occurred significantly faster (− 1%/day *versus.* - 0.6%/day; *p* = 0.03). For all other biological parameters, the relation was not significant.

#### PFS and grade of toxicity

There was no significant difference in PFS between grades of toxicity for all biological parameters.

#### PFS and highest change in percent of biological parameters

Longer PFS was correlated with an absolute one-percent increase in glycemia (*p* = 0.001) and ASAT (*p* = 0.04) (HR: 0.96 [95% CI: 0.93-0.98]; HR: 0.98 [95% CI: 0.96-0.99] respectively) and an absolute one-percent decrease in phosphatemia (*p* = 0.03) (HR: 1.04 [95% CI: 1.003-1.09]). Shorter PFS was correlated with an absolute one-percent decrease in lymphocytes (*p* = 0.004) (HR: 1.01 [95% CI: 1.005-1.02]).

For all other biological parameters, the correlation was not significant.

## Discussion

To our knowledge, this is the first study analyzing retrospectively the relationship between metabolic toxicities and mTORi efficacy in patients with mRCC. Our findings showed that a CB was more frequent when liver transaminases and serum creatinine increased, and when absolute changes in glycemia, phosphatemia, and liver transaminases were higher. A short time to the highest lymphopenia grade and a long time to the highest hyperglycemia grade were correlated with a progressive disease. The onset rate of lymphopenia was faster in progressive patients compared with those having a CB. A longer PFS was associated with an absolute one-percent increase in glycemia and ASAT and an absolute one-percent decrease in phosphatemia, whereas an absolute one-percent decrease in lymphocytes was associated with a shorter PFS. Efficacy of mTORi in this series was better than expected maybe because of young age and good performance status (ECOG-PS) of patients.

As regards hypophosphatemia and increase in liver transaminases, the incidence rates of grade 3–4 toxicities reported in pivotal phase III trials were 4% and < 1%, respectively, which were close from our findings [8, 9]. In contrast, there were some discrepancies between our results and those published in the literature. The rates of grade 3–4 hyperglycemia and lymphopenia were higher in our everolimus subgroup than those described in the literature (13% *vs.* 4%; 20% *vs.* 3%, respectively) [9] whereas the rates of grade 3–4 hyperglycemia were lower in the temsirolimus subgroup than those reported in the literature (6% *vs.* 11%) [8]. These discrepancies may be due on the one hand to the small sample size, and on the other hand to a high proportion of patients (64%) who were treated in third-line or more.

Correlations between efficacy and toxicity were highlighted in our study by using two approaches: the classical CTC-AE developed by the National Cancer Institute, and the assessment of time to highest grade of toxicity and changes in the absolute rates, correlated with RECIST tumor response (RECIST 1.1 criteria) and PFS. The classical CTC-AE scale presents several limitations as, for instance, the assessment is clinician-based and the reporting is potentially incomplete [20]. Although our findings are of interest, one should keep in mind several limitations of the study. It was a retrospective study and, even if the data were prospectively recorded, there were several missing data. No correction for multiple testing has been made and relation with a p value close to 5% should be taken cautiously. Twelve patients withdrew treatment because of toxicity before efficacy assessment: this could be responsible for modification of estimation of relationship between efficacy and toxicity. Furthermore, the lack of significant relationship can be due to the small sample size (*n* = 75). Moreover, we included patients with 6 various RCC histological subtypes, knowing that these tumors have unequal sensitivity to mTORi inducing a bias in the efficacy assessment. Finally, patients received either everolimus or temsirolimus. If both drugs have close toxicities, their frequencies are not similar. This discrepancy could induce a bias in the estimate of toxicity rate. A final potential bias in this estimate was the line of therapy: some patients received mTORi as first line (*n* = 8), whereas others received mTORi in the salvage setting (*n* = 67).

The most common toxicities of routinely used antiangiogenics (sunitinib, sorafenib) are hypertension, fatigue, hand-foot syndrome, hypothyroidism, increased lipase, and lymphopenia [21]. Everolimus and temsirolimus present with a different toxicity profile, leading especially to frequent metabolic disorders such as hyperglycemia, hyperlipidemia, and hypophosphatemia [22]. Our choice was to not analyze the correlations between clinical toxicities and efficacy of mTORi, such as pneumonitis, because its incidence was low (around 14%) in previously published data for everolimus in the RECORD-1 trial [9]. Thereby, the monitoring of metabolic toxicities seemed more relevant, because of their frequency and more reproducible assessment methods.

In a series where patients with mRCC were treated with temsirolimus or interferon, treatment benefit of temsirolimus over interferon was associated with increase in serum cholesterol [23]. We did not find this relationship in our population. Several metabolic toxicities can predict efficacy of targeted therapies and should be explored, such as proteinuria for VEGF pathways inhibitors, hyperglycemia or hyperlipidemia for mTORi [24]. We hypothesized that metabolic toxicity could be related to the efficacy because the occurrence of toxicity can reflect a higher drug exposure. An interindividual pharmacokinetic variability has been described in patients receiving everolimus in case of hepatic impairment [25]. It has been also shown that the Japanese patients presented a modified pharmacokinetics of temsirolimus, allowing a maximum tolerated dose of 15 mg/m^2^ once a week instead of 25 mg/m^2^ [26]. Besides, intra-patient dose titration is routinely performed with axitinib in order to increase axitinib exposure in patients who do not develop hypertension. Indeed, it has been shown that higher drug exposure and diastolic blood pressure were independently associated with longer PFS and OS, and with a higher probability of partial response in mRCC patients [27]. This tends to demonstrate that diastolic blood pressure could be a potential marker of efficacy. Finally, a well-described and reproducible assessment of metabolic toxicity might be a useful biomarker to predict efficacy in the drug development process [28].

Our study suggested that biological toxicities of mTORi (hypophosphatemia, hyperglycemia, and increase in liver transaminases) were significantly correlated with mTORi efficacy and lymphopenia with lack of efficacy. Given this preliminary result, it seems difficult to use a single biological parameter or a single descriptive method to predict treatment efficacy on the basis of toxicity. The relationship between a greater number of toxicities and clinical benefit should be investigated but we did not assess these relationships because of the sample size in our series.

## Conclusion

Our retrospective study suggested that hypophosphatemia, hyperglycemia, and increase in liver transaminases were significantly correlated with mTORi efficacy and lymphopenia with lack of efficacy. On the other hand, hypercholesterolemia and hypertriglyceridemia had no relationship with antitumoral efficacy of mTORi.

We noticed that the time to severe toxicity was short with a median time of 28 to 56 days, highlighting that toxicities occurred earlier than the first tumor assessment (84 days). These interesting results require to be prospectively confirmed on a larger and independent cohort of patients.

## Abbreviations

CB: Clinical benefit
CTC-AE: Common toxicity criteria for adverse events
HR: Hazard ratios
mRCC: Metastatic renal cell carcinoma
mTORi: mTOR inhibitors
OS: Overall survival
PD: Progressive disease
PFS: Progression-free survival
